# Role of reactive oxygen species and sulfide-quinone oxoreductase in hydrogen sulfide-induced contraction of rat pulmonary arteries

**DOI:** 10.1152/ajplung.00283.2016

**Published:** 2017-12-28

**Authors:** Jesus Prieto-Lloret, Vladimir A. Snetkov, Yasin Shaifta, Inmaculada Docio, Michelle J. Connolly, Charles E. MacKay, Greg A. Knock, Jeremy P. T. Ward, Philip I. Aaronson

**Affiliations:** Division of Asthma, Allergy and Lung Biology, King’s College London, London, United Kingdom

**Keywords:** hydrogen sulfide, mitochondria, protein kinase C, pulmonary artery, rat, reactive oxygen species, sulfide-quinone oxoreductase

## Abstract

Application of H_2_S (“sulfide”) elicits a complex contraction in rat pulmonary arteries (PAs) comprising a small transient contraction (phase 1; Ph1) followed by relaxation and then a second, larger, and more sustained contraction (phase 2; Ph2). We investigated the mechanisms causing this response using isometric myography in rat second-order PAs, with Na_2_S as a sulfide donor. Both phases of contraction to 1,000 μM Na_2_S were attenuated by the pan-PKC inhibitor Gö6983 (3 μM) and by 50 μM ryanodine; the Ca^2+^ channel blocker nifedipine (1 μM) was without effect. Ph2 was attenuated by the mitochondrial complex III blocker myxothiazol (1 μM), the NADPH oxidase (NOX) blocker VAS2870 (10 μM), and the antioxidant TEMPOL (3 mM) but was unaffected by the complex I blocker rotenone (1 μM). The bath sulfide concentration, measured using an amperometric sensor, decreased rapidly following Na_2_S application, and the peak of Ph2 occurred when this had fallen to ~50 μM. Sulfide caused a transient increase in NAD(P)H autofluorescence, the offset of which coincided with development of the Ph2 contraction. Sulfide also caused a brief mitochondrial hyperpolarization (assessed using tetramethylrhodamine ethyl ester), followed immediately by depolarization and then a second more prolonged hyperpolarization, the onset of which was temporally correlated with the Ph2 contraction. Sulfide application to cultured PA smooth muscle cells increased reactive oxygen species (ROS) production (recorded using L012); this was absent when the mitochondrial flavoprotein sulfide-quinone oxoreductase (SQR) was knocked down using small interfering RNA. We propose that the Ph2 contraction is largely caused by SQR-mediated sulfide metabolism, which, by donating electrons to ubiquinone, increases electron production by complex III and thereby ROS production.

## INTRODUCTION

Hydrogen sulfide (H_2_S, hereafter referred to as sulfide) typically acts as a vasodilator but in some arteries causes constriction or a complex response that exhibits both constricting and dilating phases (26). In rat pulmonary arteries (PAs), for example, application of 1 mM sulfide (as NaHS) to PAs preconstricted with norepinephrine caused a brief transient constriction followed by a relaxation and then a second and more sustained constriction (25). This response resembles the effect of hypoxia in these arteries, and based on this similarity and other observations, Olson and coworkers (25, 27) proposed that hypoxic pulmonary vasoconstriction (HPV) is due to a build-up of cellular sulfide concentration ([sulfide]) during hypoxia resulting from an inhibition of its oxidative metabolism.

Although a number of mechanisms, most notably K_ATP_ channel activation (24), have been shown to contribute to the vasodilating effect of sulfide, the pathways underlying sulfide-induced vasoconstriction are less well understood. A study in perfused trout gills (35) showed that the constriction evoked by 100 μM sulfide was strongly suppressed by blockers of mitochondrial complexes I, III, and IV and by the antioxidant diethyldithiocarbamate, leading the authors to propose a role for mitochondrially derived reactive oxygen species (ROS). It has also been reported (25) that 1 mM NaHS depolarized bovine PA smooth muscle, which would be predicted to cause contraction by opening voltage-gated Ca^2+^ channels. In addition, sulfide can activate several isoforms of protein C in rat cardiomyocytes (29), an effect that, if it occurred in PA smooth muscle cells (PASMCs), would be likely to cause contraction. Evidence has also been presented that low concentrations of sulfide constricted rat aorta and increased blood pressure by scavenging nitric oxide (2).

Sulfide has complex effects on mitochondrial respiration because it can both inhibit and stimulate the electron transport chain (ETC). The former effect, which accounts for its poisonous nature, is due to a block of cytochrome-*c* oxidase (CCOx), whereas the latter, which has been described much more recently, is associated with its metabolism by sulfide quinone oxoreductase (SQR), a process that feeds electrons into the ETC by reducing ubiquinone (12, 38). We have presented evidence in abstract form that the second sulfide-induced contraction of rat PAs is abolished by the complex III blocker myxothiazol and is associated with mitochondrial hyperpolarization, leading us to propose that it is caused by an increased flux of electrons through the distal ETC, which results in an increased generation of ROS by complex III (1, 8, 31). Here, we report that sulfide induces ROS production by PASMCs, which is ablated by SQR knockdown, and that the sustained sulfide-induced contraction is largely dependent on complex III and NADPH oxidase.

## METHODS

### 

#### Ethical approval.

This study conforms with the *Guide for the Care and Use of Laboratory Animals* published by the National Institutes of Health (NIH Publication No. 85-23, Revised 1996) and is in accordance with UK Home Office regulations [Animals (Scientific Procedures) Act, 1986; King's College London Animal Welfare Assurance number F17-00373]. Male Wistar rats (200–300 g; 6–8 wk old) were euthanized by lethal injection (intraperitoneal) of sodium thiopental.

#### Intrapulmonary artery mounting and measurement of tension development.

The heart and lungs were excised and placed in cold physiological salt solution (PSS) that contained the following (in mmol/l): 118 NaCl, 24 NaHCO_3_, 1 MgSO_4_, 0.435 NaH_2_PO_4_, 5.56 glucose, 1.8 CaCl_2_, and 4 KCl. Rings of the intrapulmonary artery (IPA; inner diameter: 0.5−1.0 mm) were dissected free of adventitia and parenchyma under a dissection microscope, mounted on a conventional small vessel wire myograph, and stretched to give a basal tension of ~5–6 mN (equivalent to an internal pressure of ~15 mmHg). They were then equilibrated with three brief exposures to PSS containing 80 mmol/l KCl (80KPSS; isotonic replacement of NaCl by KCl).

#### ROS measurements.

Rat PASMCs cultured to passage 4 were transfected using Amaxa electroporation kit with plasmid encoding either scrambled small interfering (si)RNA or SQR siRNA, seeded in white 96-well plates and growth arrested for 24 h. Culture medium was then replaced with warm (37°C) Krebs solution gassed with air-5% CO_2_ containing 10 µM L-012. Basal luminescence was measured with a Promega plate reader, solution in wells was replaced with Krebs containing 1,000 μM Na_2_S, and the plate was incubated at 37°C for 10 min. Cells were then washed twice with Krebs, 10 µM L-012 were added, and luminescence was measured again. As the L-012 signal without cells was <1% of that with cells, no background subtraction was necessary. Results were expressed as signal increase over control (i.e., cells not treated with Na_2_S) in each well, and average values for control and SQR-silenced cells were compared. Separate control experiments have shown that incubation without Na_2_S caused no significant changes in luminescence.

#### Estimation of changes in NAD(P)H.

Changes in NAD(P)H levels were assessed as described previously (22). Briefly, the autofluorescence of IPA mounted on a myograph was recorded while the preparation was alternately excited with light at 340 and 380 nm while fluorescence at 500 nm was recorded. This approach depends on the fact that NAD(P)H fluorescence at ∼500 nm is greater when excited at 340 nm than at 380 nm, such that the 340/380 emission ratio is proportional to changes in NAD(P)H. Movement of the preparation does not affect the ratio as it equally affects emission at both wavelengths.

#### Estimation of mitochondrial membrane potential.

PAs were mounted in a confocal wire myograph (DMT) set on the stage of inverted microscope combined with a microspectrofluorimeter (Cairn). Arteries were stretched and stimulated with 80KPSS as described above and then incubated in PSS containing 1 μM tetramethylrhodamine ethyl ester (TMRE) for ~30 min. The artery was then washed several times with dye-free PSS, and TMRE emission at >530 nm was recorded using an excitation wavelength of 490 nm, together with force. Experiments were started after the TMRE signal had stabilized. This method was based on the protocol described in the Molecular Probes handbook (Section 22.3, see https://www.thermofisher.com/uk/en/home/references/molecular-probes-the-handbook/probes-for-membrane-potential.html) and depends on the concept that when TMRE is used in relatively high concentrations, an increase in its mitochondrial concentration (which occurs during mitochondrial hyperpolarization since TMRE is positively charged) will cause a fall in fluorescence due to self-quenching; likewise fluorescence will increase upon mitochondrial depolarization (3, 18, 21). We validated this approach in preliminary experiments by showing that FCCP, cyanide, and antimycin all caused an increase in TMRE fluorescence. Severe hypoxia also caused an increase in fluorescence that was reversible upon reimposition of normoxia (not shown).

#### PCR.

RNA was isolated from homogenates of freshly isolated rat PAs, mesenteric arteries, aorta, brain, and cultured PASMCs. Reverse transcription of the RNA was carried out as previously described (16). RT-PCR primer pairs for rat sulfide quinone reductase-like (SQRDL) were designed using Primer3web version 4.0 (http://primer3.ut.ee) and synthesized by Sigma-Aldrich (St. Louis, MO). SQRDL (Accession No. BC158559) primers were sense CTGCAGGACTTCAAGGAAGG and antisense CTCTCCCGAATGATCTCCTG. PCR was carried out using PuReTaq Ready-To-Go PCR Beads (GE Healthcare, Piscataway, NJ), and the PCR products (reaction equivalent on 20 ng reverse transcribed RNA) were analyzed by electrophoresis on 2.8% agarose gels run in TAE buffer (National Diagnostics, Hessle, UK) with PhiX174 DNA/HinfI Marker (Thermoscientific, Waltham, MA).

#### Cell culture, siRNA design, and cell transfection.

PASMCs and mesenteric artery smooth muscle cells were dispersed from PAs and mesenteric arteries, respectively, using collagenase (type XI, 2 mg/ml), trypsin inhibitor (1 mg/ml), and papain (1 mg/ml), and cultured in DMEM containing 10% FCS as previously described (37). PASMCs from passages 3–4 were growth arrested in serum-free medium for 24 h before use; each cell line was verified as smooth muscle by immunostaining for smooth muscle α-actin, calponin, and desmin (Sigma-Aldrich).

Several siRNAs against SQR were designed as described previously (16) and tested for knock-down efficiency, and the best were selected for experimental use. The 19 nucleotide target sequences (SQRDL-siRNA: position 884–902, GenBank Accession No. BC158559) were synthesized into 64–65 mer oligonucleotides with BamHI/HindIII overhangs (Sigma-Aldrich) and cloned into the expression vector pSilencer 3.0-H1 (Life Technologies, Paisley, UK). The siRNA clone was purified using an EndoFree Plasmid Maxi Kit (Qiagen, Crawley, UK) and sequenced (Geneservice, Cambridge, UK). PASMCs were transfected using the Basic Nucleofector Kit for Primary Mammalian Smooth Muscle Cells and a nucleofector device (Nucleofector Technology, Lonza, Slough, UK); after 72 h cells were serum starved for 24 h before use. Transfection efficiency was >80%, as determined using pmaxGFP (green fluorescent protein expressing vector) provided in the kit and confirmed by fluorescence microscopy. Efficiency and selectivity of knockdown were confirmed by Western blot.

#### Measurement of [sulfide].

The [sulfide] in PSS was measured and recorded using a hydrogen sulfide amperometric sensor and an amplifier and SensorTrace Suite software from Unisense (Aarhus, Denmark).

#### Application of sulfide.

Sulfide was applied to the myograph chamber by adding aliquots of Na_2_S (500 mM dissolved in PSS) to the solution. Final Na_2_S concentrations ranged from 10 to 1,000 μM. Whereas application of 10–100 μM Na_2_S to the solution had no effect on pH, higher concentrations caused small but transient rise in pH. In particular 300, 500, and 1,000 μM caused an increase in pH, which peaked at ~10 s (increases of 0.06, 0.1, and 0.2 units, respectively) and then progressively diminished; at 60 s the pH was elevated by 0.04, 0.06, and 0.1 pH units, and at 3 min 0.02, 0.02, and 0.03 pH units, respectively. By 5 min, the pH had returned to the level recorded before Na_2_S was applied.

#### RhoA activation.

PA segments were incubated for 20 min in Eppendorf tubes containing PSS or for 10 min in PSS and then 20 min in PSS containing 500 μM Na_2_S. Tubes were gassed continually with air/CO_2_. Tissues were then snap frozen and protein extracted as previously described (16). Activation of RhoA was assessed using a RhoA G-LISA Activation Assay (luminescence format) from Cytoskeleton (Denver, CO) as described by the manufacturer’s instructions.

#### Data analysis and statistics.

Data were analyzed using Student’s paired or unpaired *t*-test as appropriate. The threshold for statistical significance for all comparisons was set at *P* < 0.05. GraphPad Prism was used for all analyses. Summary data values shown in the figures are the means ± SE. In experiments in which the fall of the [sulfide] in the myograph chamber was recorded following its injection into the solution, this process was closely approximated by a monoexponential function after the concentration had fallen by ~60% from its peak. This portion of the curve was therefore fitted using the following equation: [sulfide]_t_ = [sulfide]_0_ × *e*^−^*^kt^* + *a*, where *a* is constant of ≥ 0, *t* is time, and *k* is the rate constant of the exponential decay.

## RESULTS

### 

#### Sulfide induces a biphasic contraction in rat IPAs.

**Fig. 1.**
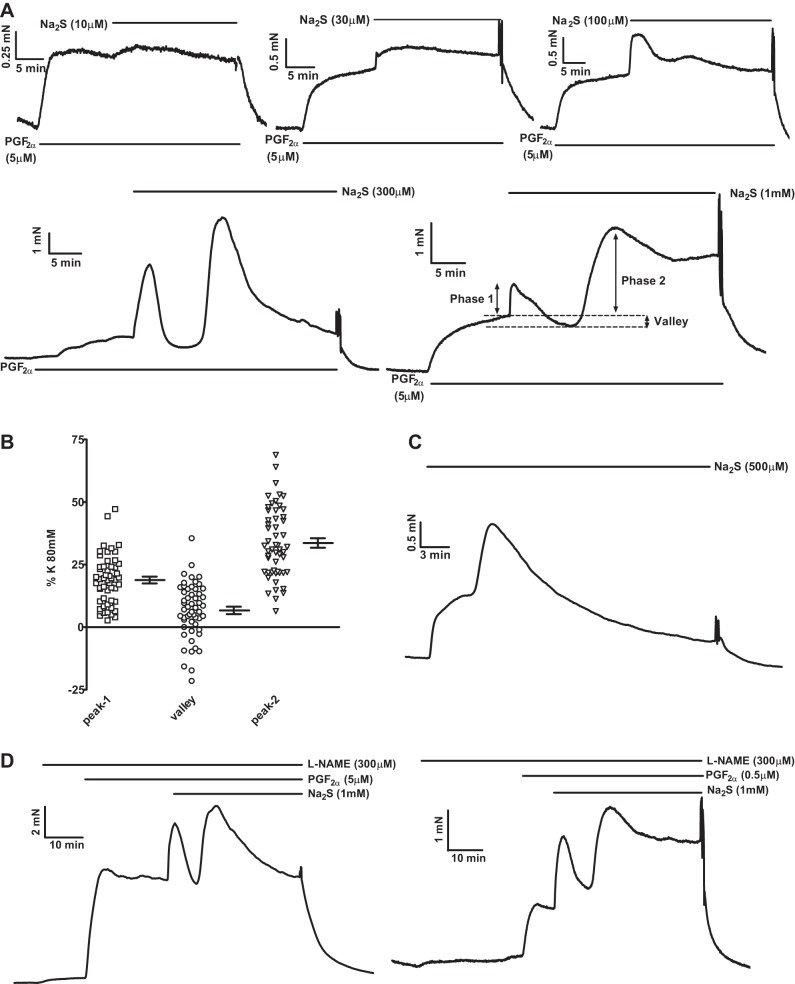
Properties of sulfide-induced contraction in rat pulmonary arteries (PAs). *A*: representative examples of contractile responses evoked by 10–1,000 μM Na_2_S in the presence of 5 μM PGF_2α_. *B*: scatterplot showing the amplitudes of the 3 phases of the response to 1,000 μM Na_2_S in PGF_2α_ in 54 individual PAs; means ± SE values are shown at the *right* of each set of points. *C*: effect of 500 μM Na_2_S in the absence of PGF_2α_. *D*: representative responses to 1,000 μM Na_2_S in the presence of 300 μM nitro-l-arginine methyl ester (l-NAME) with preconstriction induced by 0.5 (*left*) or 5 μM (*right*) PGF_2α_.

**Fig. 2.**
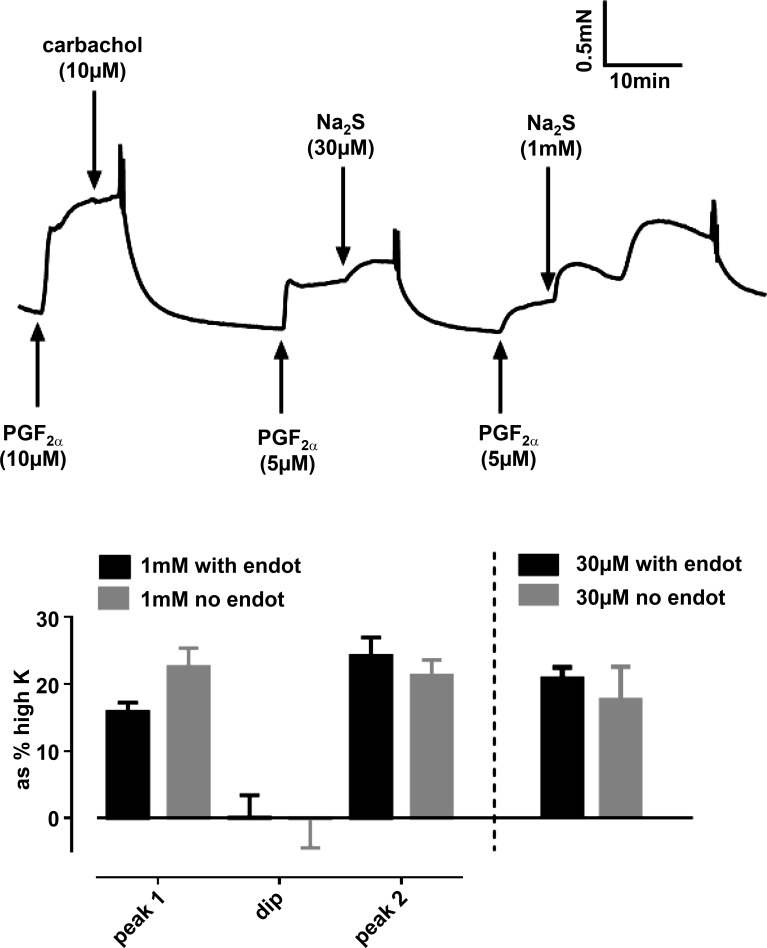
Lack of effect of endothelial denudation on the contraction to Na_2_S The trace shows an example of contractile responses to 30 μM and 1,000 μM Na_2_S, both applied in the presence of 5 μM PGF_2α_, in a pulmonary artery denuded of endothelium (note the absence of vasorelaxation to 10 μM carbachol). The bar chart (*left*) presents the means ± SE amplitudes of the 3 phases of the response to 1,000 μM Na_2_S in arteries in which the endothelium was left intact (black bars; *n* = 13) or denuded (gray bars; *n* = 20). The contractile responses to 30 μM Na_2_S in endothelium-intact (*n* = 30) and -denuded (*n* = 9) arteries are shown on the *right*. None of the responses were significantly different between endothelium-intact and -denuded arteries.

Contractions evoked by Na_2_S applied on its own were variable, with many tissues showing no or very small responses, especially at concentrations <500 μM. However, if arteries were first slightly preconstricted with a low concentration of PGF_2α_, consistent responses were observed at sulfide concentrations of ≥10 μM (Fig. 1*A*). At low concentrations (10 and 30 μM), sulfide caused a small monophasic contraction, whereas a complex response occurred at higher concentrations (100–1,000 μM). This consisted of an initial brief contraction [phase 1 (Ph1)], followed by a relaxation and then a second increase in tension [phase 2 (Ph2)], which waned with time. The amplitude of the three components of the response evoked by 1,000 μM Na_2_S, measured at the peaks of the two contractions and the nadir of relaxation and expressed as the change in tension from that recorded in 5 μM PGF_2α_ just before sulfide application, is illustrated in Fig. 1*B*. Note that tension during the relaxation observed between the two contractions often fell below the preconstriction level, indicating that this involved an active vasorelaxation rather than merely the waning of the initial contraction. Figure 1*C* illustrates a typical response to sulfide applied on its own (i.e., in the absence of preconstriction), which was observed in some arteries; under these conditions responses most commonly demonstrated, an initial shoulder followed by a larger contraction that reached a peak and then decayed. As depicted in Fig. 1*D*, *left*, a typical response to Na_2_S in the presence of 5 μM PGF_2α_ was observed when the endothelial nitric oxide synthase inhibitor nitro-l-arginine methyl ester (l-NAME; 300 μM) was applied. Because l-NAME tended to increase PGF_2α_-induced preconstriction, and this might have artifactually increased the response to sulfide, we carried out similar experiments using a lower concentration of PGF_2α_ (0.5 μM), which gave a level of preconstriction similar to that observed in 5 μM PGF_2α_ in the absence of l-NAME, and again saw the usual response to sulfide (Fig. 1*D*, *right*). The responses to both 30 and 1,000 μM sulfide were also not significantly affected by endothelial denudation (Fig. 2).

#### Contraction induced by sulfide is temporally associated with changes in NAD(P)H autofluorescence.

**Fig. 3.**
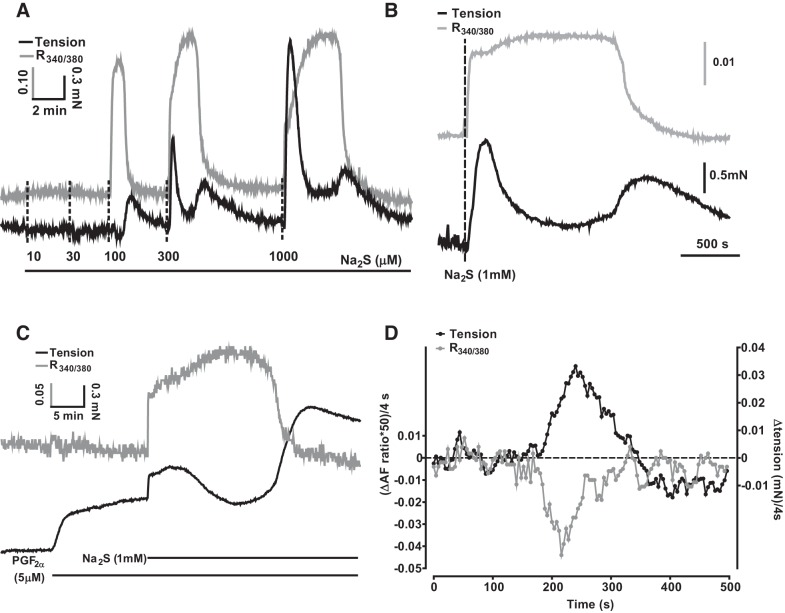
Effects of sulfide on NAD(P)H autofluorescence. *A*: representative experiment showing simultaneous recordings of the effect of 10–1,000 μM Na_2_S on the NAD(P)H/NADP autofluorescence ratio (gray trace) and tension (black trace) in the absence of PGF_2α_. *B* and C: representative experiments highlighting the close temporal relationship between the 2 phases of the contraction and the onset/offset of the increase in the NAD(P)H/NADP autofluorescence ratio evoked by Na_2_S in the absence (*B*) and presence (*C*) of PGF_2α_ (5 μM). *D*: rates of change of the tension and autofluorescence signals (each recorded at 4-s intervals) from the experiment illustrated in *B* over a 500-s period encompassing the onset of the phase 2 (Ph2) contraction. Traces were smoothed by calculating the running average of the values over 5 points (i.e., for the tension at time = 200 s, this was the average of the values recorded at 192, 196, 200, 204, and 208 s), and then the differences between the resulting smoothed values at each successive point (i.e., that at point 200 s minus that at 196 s) were plotted vs. time. The autofluorescence was scaled (×50) to facilitate visual comparison with the tension (note the scale bars in *B*).

Since sulfide blocks CCOx, we explored the role of the mitochondria in the response to sulfide by recording the NAD(P)H/NAD(P) ratio, as an indication of block of the ETC, and simultaneously measured contraction. As shown in Fig. 3*A*, application of a range of concentrations of sulfide caused a rapidly developing and transient increase in the NAD(P)H/NAD(P) ratio, which became apparent at a concentration of 100 μM sulfide and increased in duration as a function of the sulfide concentration. As shown more clearly in Fig. 3, *B* and *C*, in both the absence (Fig. 3*B*) and presence (Fig. 3*C*) of PGF_2α_ the Ph1 of contraction began just after the onset of the change in autofluorescence, whereas the nadir of the contraction occurred during the period when the NAD(P)H/NADP ratio was maximal.

The subsequent onset of the Ph2 contraction coincided closely with the rapid decline of the NAD(P)H/NADP autofluorescence signal to its baseline level. To analyze the temporal overlap of these two events, we calculated the rate of change (i.e., the slopes) of both signals during the relevant period by taking the difference between the amplitude of each signal at each time point and the time point immediately before it, such that increasing contraction would result in a positive slope value for the tension signal and falling autofluorescence would yield a negative slope value for this signal. Figure 3*D* depicts the slopes of both signals over a 500-s period encompassing the onset of Ph2 of a contraction induced by 300 μM sulfide. It was apparent that the periods during which the signals were changing overlapped closely.

Moreover, the slopes of both signals exhibited maxima that occurred at similar times (Fig. 3*D*). In nine experiments of this type, the slope of the Ph2 contraction reached its maximum 7 ± 11 s before the NAD(P)H/NADP autofluorescence signal reached its maximum slope, indicating that the reversal of the sulfide-induced increase in autofluorescence and the Ph2 contraction occurred almost simultaneously. The fall in the autofluorescence signal presumably represented unblock of the CCOx and the ETC; this would be predicted to occur once the sulfide concentration in the solution, which falls continually following Na_2_S application as H_2_S is lost to the atmosphere (see Fig. 8 below, and also Ref. 17), reaches a low enough level.

#### Contraction induced by sulfide is also temporally associated with hyperpolarization of the mitochondrial membrane potential.

**Fig. 4.**
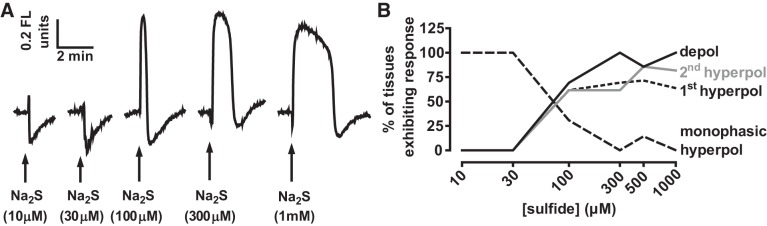
Effects of different concentrations of sulfide on ΔΨ. *A*: effects of 10–1,000 μM Na_2_S on ΔΨ. Depolarization and hyperpolarization of ΔΨ are indicated by up and downward displacement of the traces, respectively. *B*: percentage of arteries demonstrating a depolarization (depol), a hyperpolarization preceding the depolarization (1st hyperpol), a hyperpolarization following the depolarization (2nd hyperpol), or a hyperpolarization in the absence of a depolarization (monophasic hyperpol) following the application of a number of concentrations of Na_2_S. Arteries demonstrated only hyperpolarization to 10 and 30 μM sulfide. At higher concentrations of Na_2_S, arteries mostly demonstrated a triphasic response (see responses to 300 μM and 1 mM Na_2_S in *A*) but in some cases one of the phases was not observed (e.g., the 1st hyperpolarization is absent in the response to 100 μM Na_2_S shown in *A*). The number of arteries studied at each concentration ranged between 7 and 17.

We also examined the effect of different concentrations of sulfide on the mitochondrial membrane potential (ΔΨ) using TMRE. As shown in Fig. 4*A*, low concentrations of sulfide (10 and 30 μM) invariably caused a mitochondrial hyperpolarization (*n* = 11 and 17, respectively). In contrast, ≥300 μM sulfide almost invariably (30/31 arteries) caused a depolarization. However, in most arteries (21/31), this was preceded by a brief hyperpolarization, and followed by a more prolonged hyperpolarization (23/31). The intermediate concentration of 100 μM sulfide caused a monophasic hyperpolarization in some tissues (4/13), while the remainder demonstrated the type of triphasic response usually observed with ≥300 μM sulfide. The frequency with which these effects on ΔΨ were observed at each sulfide concentration is shown in Fig. 4*B*.

**Fig. 5.**
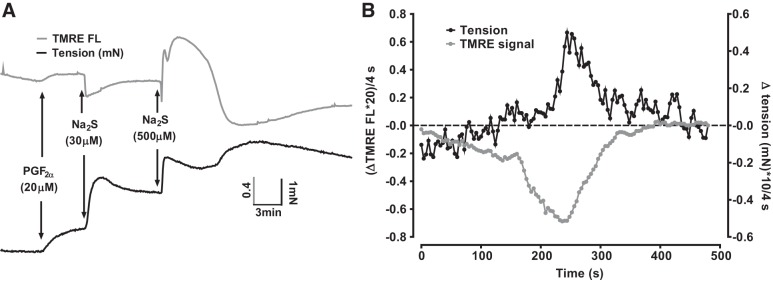
Simultaneous recording of the effect of 30 and 500 μM sulfide on ΔΨ and constriction *A*: representative experiment showing the simultaneous recording of ΔΨ [tetramethylrhodamine ethyl ester (TMRE) signal; gray trace and tension development (black trace) during the application of 30 μM and then 500 μM Na_2_S. Twenty micromolar PGF_2α_ was applied before sulfide to magnify the sulfide-induced contraction. *B*: rates of change of the tension and TMRE signals (each recorded at 4-s intervals) from the experiment illustrated in panel A over a 500-s period encompassing the development of Ph2 of contraction. Traces were smoothed by calculating the running average of the values over 5 points (i.e., for the tension at time = 200 s, this was the average of the values recorded at 192, 196, 200, 204, and 208 s), and then the differences between the resulting values at each successive point (i.e., that at point 200 s minus that at 196 s) were plotted vs time; both signals were scaled to facilitate their visual comparison.

Figure 5*A* illustrates an experiment in which the effects of applying 30 and 500 μM sulfide on ΔΨ and tension were monitored simultaneously. As in Fig. 1, PGF_2α_ was first applied to amplify the sulfide-induced contraction; in these experiments, a somewhat higher concentration of PGF_2α_ (20 rather than 5 μM) was used to further magnify the size of the contraction. This often caused a small depolarization of ΔΨ, which we did not study further. The onset of the contraction induced by 30 μM sulfide occurred simultaneously with a hyperpolarization of ΔΨ. Application of 500 μM sulfide then caused a typical triphasic response of ΔΨ. The initial hyperpolarization of ΔΨ coincided with the Ph1 of contraction. Tension then fell as ΔΨ depolarized, only to rise again as this depolarization gave way to a prolonged hyperpolarization.

The close temporal correlation between the onset of the Ph2 contraction and the hyperpolarization of ΔΨ apparent in Fig. 5*A* was observed in 10 arteries. We analyzed the rates of change of both the tension and ΔΨ signals in these arteries using the approach described above for Fig. 3*D*. Figure 5*B* illustrates the slopes of both signals over a 500-s period beginning ~200 s before the development of the Ph2 contraction induced in an artery by the application if 500 μM sulfide. Both signals changed over a similar period. On average, the contraction reached its maximum rate of increase 35 ± 11 s (*n* = 10) after the point at which the rate of ΔΨ hyperpolarization was maximal.

In addition to blocking CCOx, which would be predicted to depolarize the mitochondria, at lower concentrations sulfide is also able to stimulate mitochondrial electron flow as a result of its metabolism by SQR, since during this process SQR is reduced and passes electrons into the ETC via ubiquinone (38). We reasoned that the complex effect of sulfide on ΔΨ might be due to the superimposition of ΔΨ depolarization caused by block of CCox on ΔΨ hyperpolarization due to SQR-mediated stimulation of the ETC.

In this case, it would be predicted that the hyperpolarization of ΔΨ caused by 30 μM sulfide, and also the two phases of hyperpolarization observed in the presence of higher concentrations of sulfide, would be preserved in the presence of the complex 1 blocker rotenone, since electrons from SQR enter the ETC distal to complex 1 (38).

**Fig. 6.**
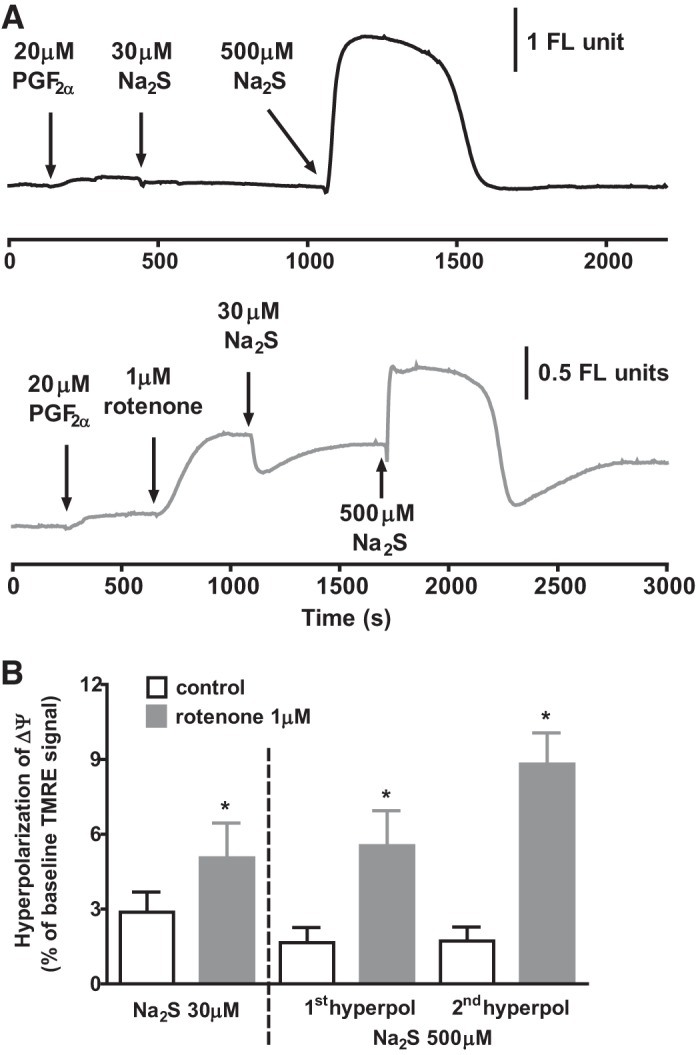
Effect of sulfide on ΔΨ in the absence and presence of rotenone. *A*, *top trace*: effect of applying 30 and then 500 μM Na_2_S on ΔΨ. *A*, *bottom trace*: mitochondrial membrane potential during the application of the same concentrations of Na_2_S following the prior application of 1 μM rotenone, which caused a rapid depolarization of on ΔΨ. *B*: means ± SE hyperpolarization of ΔΨ by 30 and 500 μM Na_2_S in the presence and absence of rotenone. Bars represent the peak amplitudes of the monophasic hyperpolarization of ΔΨ recorded in 30 μM Na_2_S and of the 2 phases of hyperpolarization recorded in 500 μM Na_2_S (see example in *A*) in the presence and absence of rotenone in groups of tissues (*n* = 15 and 9 for controls and rotenone-treated arteries exposed to 30 μM Na_2_S, respectively; *n* = 6 for both groups exposed to 500 μM Na_2_S). *P < 0.05, rotenone vs. control by unpaired *t*-test.

As shown in Fig. 6*A*, both the monophasic hyperpolarization of ΔΨ evoked by 30 μM sulfide and the two hyperpolarizing phases of the response to 500 μM sulfide were still present in 1 μM rotenone and were in fact significantly increased in amplitude. Mean results from a number of experiments in which the effects of 30 and 500 μM sulfide on ΔΨ recorded the presence and/or absence of rotenone are shown in Fig. 6*B*.

In contrast to the effect of inhibiting complex 1, it would be predicted that blocking the ETC distal to ubiquinone should prevent sulfide-induced hyperpolarization of ΔΨ, since this would effectively stop the forward flow of electrons through the ETC. We therefore examined the effect of myxothiazol, which inhibits the entry into complex 3 of electrons from ubiquinone.

**Fig. 7.**
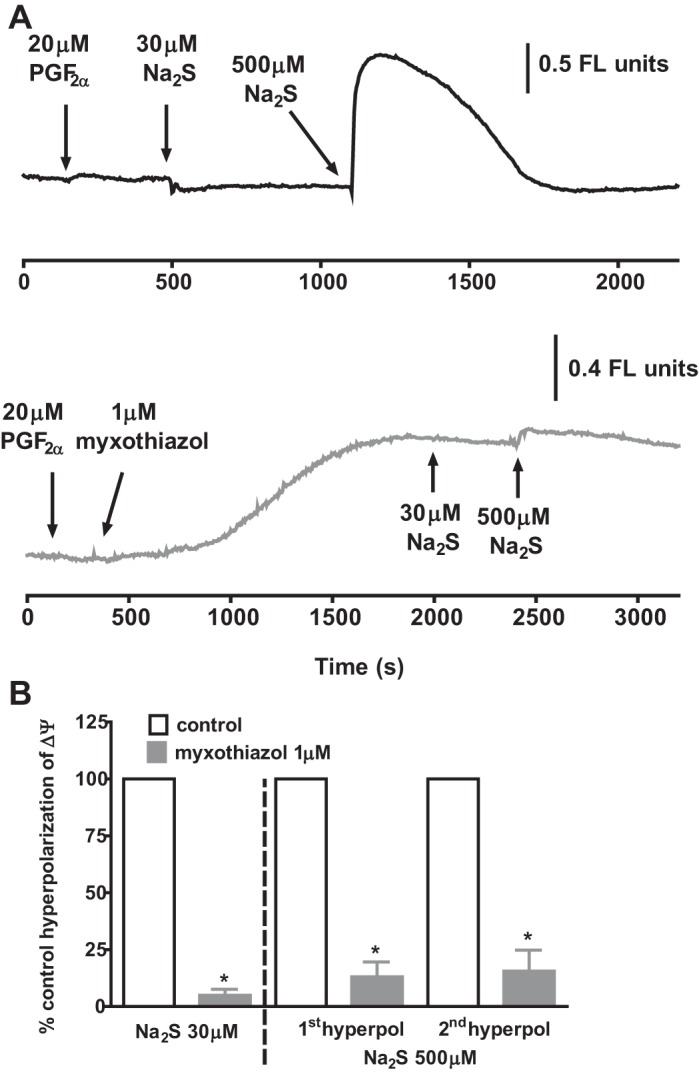
Effect of sulfide on ΔΨ in the absence and presence of myxothiazol. *A*, *top trace*: effect of applying 30 and then 500 μM Na_2_S on ΔΨ. *A*, *bottom trace*: mitochondrial membrane potential during the application of the same concentrations of Na_2_S following the prior application of 1 μM myxothiazol, which caused a slow depolarization of on ΔΨ. *B*: Bars represent the means ± SE of the peak monophasic hyperpolarization of ΔΨ by 30 μM Na_2_S (*left*) and the 1st and 2nd hyperpolarizations of ΔΨ induced by 500 μM Na_2_S in the presence of myxothiazol, each expressed as a percentage of that previously recorded in the same tissue under control conditions (*n* = 4) or in the presence of rotenone (*n* = 1). *P > 0.05, myxothiaziole vs control or rotenone by paired *t*-test (*n* = 5).

In line with this prediction, both the monophasic repolarization induced by 30 μM sulfide and the two phases of hyperpolarization caused by 500 μM sulfide were virtually abolished by myxothiazol (Fig. 7*A*). A similar blocking effect of myxothiazol occurred if it was applied after sulfide-induced hyperpolarization of ΔΨ was first recorded in the presence of rotenone. The mean effects of myxothiazol on sulfide-induced hyperpolarization of ΔΨ are shown in Fig. 7*B*.

#### Simultaneous measurements of bath [sulfide] and contraction.

**Fig. 8.**
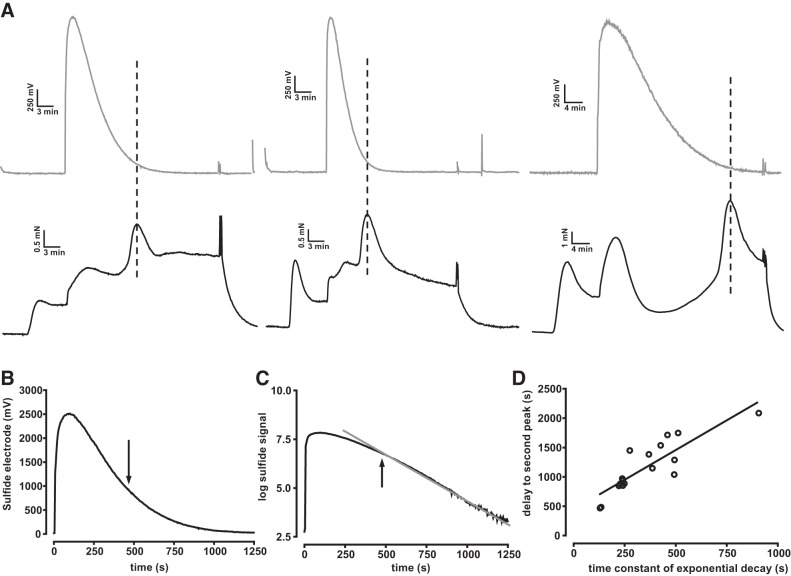
Temporal relationship between bath sulfide concentration and phase 2 (Ph2) contraction. *A*: simultaneous recording of the sulfide sensor signal (gray traces) and tension development (black traces) from 3 experiments following the injection of 1,000 μM Na_2_S into the bath. Dashed lines indicate the time at which the Ph2 contractions reached their peaks. *B* and *C*: representative sulfide sensor signal plotted using linear (*B*) and logarithmic (*C*) ordinates, highlighting the monoexponential decay of the sensor signal after it had fallen by ~60%. *D*: correlation between the time constant of the monoexponential component of decay of the sensor signal and the delay between the injection of sulfide and the peak of the Ph2 contraction.

The results described in Figs. 5–7 imply strongly that the Ph2 contraction to high concentrations of sulfide developed as block of the ETC gave way to its stimulation. We hypothesized that this was occurring when the sulfide concentration in the bath, which falls continuously following the addition of Na_2_S owing to H_2_S outgassing (28), reaches a level low enough for block of CCOx to be relieved. In this case, it would be expected that the Ph2 contraction would occur sooner if the sulfide concentration in the solution decayed more rapidly. We tested this by recording the sulfide concentration in the organ bath while monitoring the contraction. Arteries were preconstricted with PGF_2α_ to magnify the contraction, 1,000 μM Na_2_S were applied to the solution, and the rate at which the [sulfide] fell was varied between experiments by gassing the solution in the myograph chamber more or less vigorously. Figure 8*A* illustrates the results of three experiments of this type and demonstrates that the delay before the second contraction was shorter when the [sulfide] fell more rapidly. The [sulfide] at the time of the peak of the Ph2 contraction was similar in each experiment, even though the time it took for the concentration to fall to this level varied widely. In 12 experiments of this type, the mean [sulfide] measured at the peak of the second contraction ranged from 36 to 55 μM, with an average value of 49 ± 1.4 μM. The fall in the sulfide signal was closely approximated by a single exponential once the concentration had fallen by ~60% from the peak (Fig. 8, *B* and *C*); this delay probably occurred due to mixing effects immediately after injection of sulfide and also because the sensor response is nonlinear at concentrations >300 μM sulfide. As shown in Fig. 8*D*, there was a good correlation between the time constant of this exponential phase and the time at which the peak of the second response was recorded.

It is noteworthy that we were not able to detect a measureable rise in [sulfide] following application to the solution of a high concentration (1,000 μM) of the slow-release sulfide donor GYY4137 (*n* = 6, not shown). This is probably because the rate at which sulfide was outgassing from the solution was faster than its rate of release from GYY4137.

#### Contractile pathways involved in the response to sulfide.

**Fig. 9.**
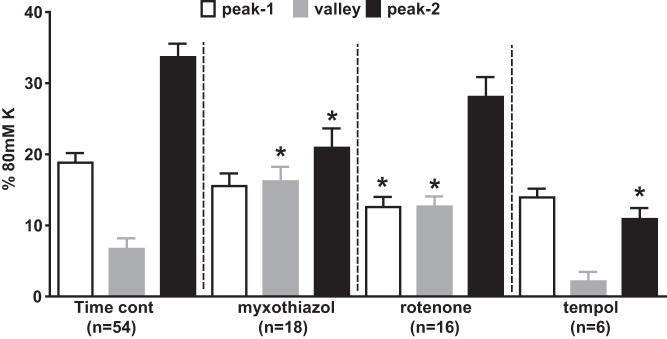
Effects of mitochondrial blockers and TEMPOL on the contractile response to 1,000 μM Na_2_S. *A*: amplitudes of the peak of the Ph1 contraction (white bars), the nadir of the subsequent relaxation (gray bars), and peak of the Ph2 contraction evoked by 1,000 μM Na_2_S in pulmonary arteries preconstricted with 5 μM PGF_2α_ under control conditions (*left*) and in the presence of myxothiazol (1 μM), rotenone (1 μM), or TEMPOL (3 mM). Numbers of replicates under each condition are shown. *P < 0.05, significantly different from the corresponding control value for that phase.

Figure 9 presents the amplitudes of the three phases of the response to 1,000 μM Na_2_S in the presence of 5 μM PGF_2α_ under control conditions (same data as in Fig. 1*C*, expressed as means ± SE) and also in the presence of rotenone (1 μM), myxothiazol (1 μM), and the antioxidant TEMPOL (3 mM). Rotenone slightly attenuated the Ph1 contraction, and abolished the subsequent vasorelaxation, but had no effect on the Ph2 contraction. Myxothiazol, which also suppressed the vasorelaxation, had no effect on the initial contraction but strongly inhibited the Ph2 contraction. TEMPOL also suppressed the Ph2 contraction.

**Fig. 10.**
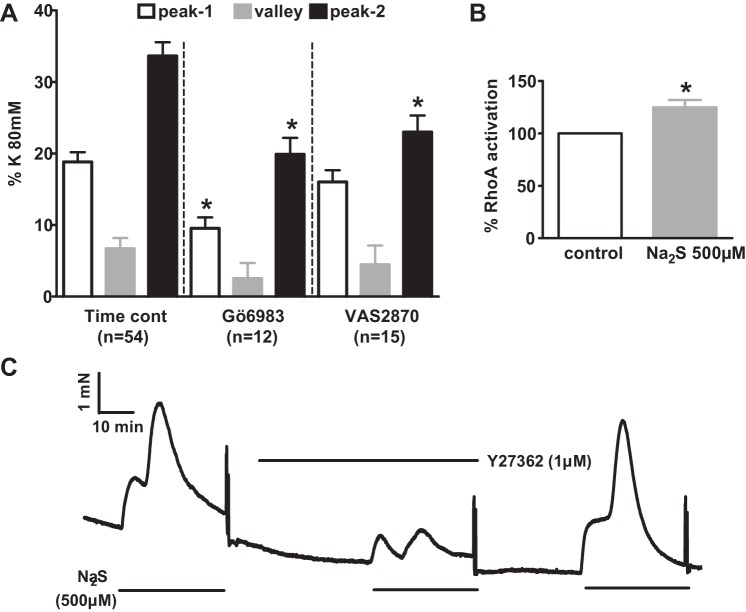
Effects of PKC, Rho kinase (ROCK), and NADPH oxidase (NOX) inhibitors on the contractile response to 1,000 μM sulfide. *A*: amplitudes of the peak of the phase 1 (Ph1) contraction (white bars), the nadir of the subsequent relaxation (gray bars), and peak of the phase 2 (Ph2) contraction evoked by 1,000 μM Na_2_S in PAs preconstricted with 5 μM PGF_2α_ under control conditions (*left*) and in the presence of Gö6983 (3 μM) or VAS2870 (10 μM). *B*: effect of 10 min incubation in 500 μM Na_2_S on RhoA activation, normalized to paired control tissues. *C*: effect of 1 μM Y27632 on the contractile response to 500 μM Na_2_S. *P < 0.05, significantly different from the corresponding control value for that phase.

Figure 10 compares the response to 1,000 μM Na_2_S in the presence of 5 μM PGF_2α_ under control conditions to that observed in the presence of the broad spectrum PKC antagonist Gö6983 (3 μM) and of the NADPH oxidase inhibitor VAS2870 (10 µm). We also examined the effect of 1 μM Y27632 [Rho kinase (ROCK) inhibitor], in this case using arteries that responded to sulfide in the absence of PGF_2α_ (since Y27632 markedly inhibits the response to PGF_2α_). As shown in Fig. 10*C*, the peak response to 500 μM sulfide was greatly decreased by Y27632. As these results indicated the possible involvement of the RhoA/ROCK pathway in the sulfide-induced response, we also investigated whether sulfide activated RhoA using a G-LISA assay, which measures GTP-bound RhoA. The assay was carried out using arterial segments that were exposed to PSS in the presence or absence of sulfide for 20 min and then snap frozen and homogenized. As depicted in Fig. 10*B*, 500 μM sulfide caused a small but significant increase in RhoA activation.

**Fig. 11.**
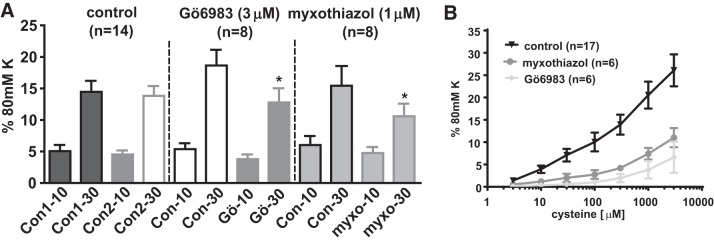
Effects of Gö6983 and myxothiazol on contractions to 10 and 30 μM Na_2_S. *A*: Amplitude of contractions evoked by two successive applications of 10 or 30 μM Na_2_S in the presence of 5 μM PGF_2α_ under control conditions (time controls) and when Gö6983 (3 μM) or myxothiazol (1 μM) was added to the bath 30 min before the second application of sulfide. *P < 0.05, significant difference between the contractions observed during the 1st and 2nd applications of sulfide. *B*: concentration-dependent contraction caused by the sulfide “donor” l-cysteine in PGF_2α_-preconstricted arteries under control conditions and in the presence of Gö6983 (3 μM) or myxothiazol (1 μM). *P < 0.05, significantly different from the corresponding control value for that phase.

Figure 11*A* shows that application of 1 μM myxothiazol or 3 μM Gö6983 inhibited the response to 30 μM sulfide, which was observed in the presence of 5 μM PGF_2α_, although neither drug significantly affected the contraction evoked by a lower sulphide concentration (10 μM).

Application of the sulfide precursor cysteine to PGF_2α_-preconstricted PAs caused a concentration-dependent contraction (see Ref. 30), which was also greatly attenuated by myxothiazol or Gö6983 (Fig. 11*B*).

**Fig. 12.**
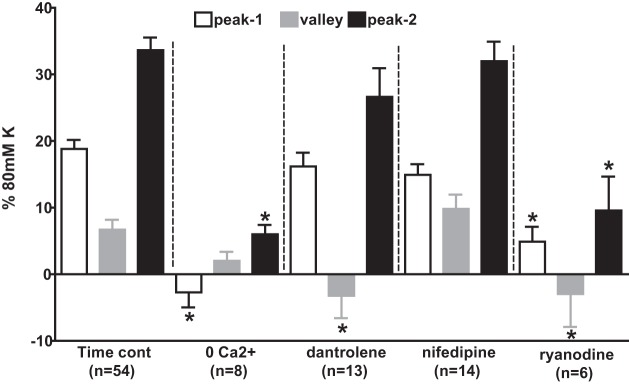
Effects of Ca^2+^ free solution, nifedipine, ryanodine, and dantrolene on the contractile response to 1,000 μM Na_2_S. Bars indicate the amplitudes of the peak of the phase 1 (Ph1) contraction (white bars), the nadir of the subsequent relaxation (gray bars), and peak of the phase 2 (Ph2) contraction (black bars) evoked by 1,000 μM Na_2_S in PAs preconstricted with 5 μM PGF_2α_ under control conditions (*left*), in Ca^2+^ free solution containing 200 μM EGTA, and in the presence of 30 μM dantrolene, 1 μM nifedipine, or 50 μM ryanodine. Numbers of replicates under each condition are shown. *P < 0.05, significantly different from the corresponding control value for that phase.

Figure 12 compares the response to 1,000 μM sulfide in the presence of 5 μM PGF_2α_ under control conditions to that seen in Ca^2+^ free solution (containing 200 μM EGTA) and in the presence of the voltage-gated Ca^2+^ channel antagonist nifedipine (1 μM), or the ryanodine receptor (RyR) blockers ryanodine (50 μM) or dantrolene (30 μM). Whereas nifedipine had no significant effect on the response to sulfide, Ca^2+^ removal and ryanodine suppressed both phases of contraction. Ryanodine also deepened the nadir of the relaxation phase and dantrolene had the same effect, although it did not significantly affect either phase of contraction.

Further experiments (not illustrated) demonstrated that TEMPOL inhibited the contractile response to 30 μM sulfide in the presence of 5 μM PGF_2α_ by 59 ± 9% (*n* = 9, *P* < 0.05), whereas in contrast ryanodine had no effect on this response (12 ± 14% inhibition, *n* = 11, ns).

#### Effect of SQR knockdown on sulfide-induced ROS production in cultured PASMCs.

**Fig. 13.**
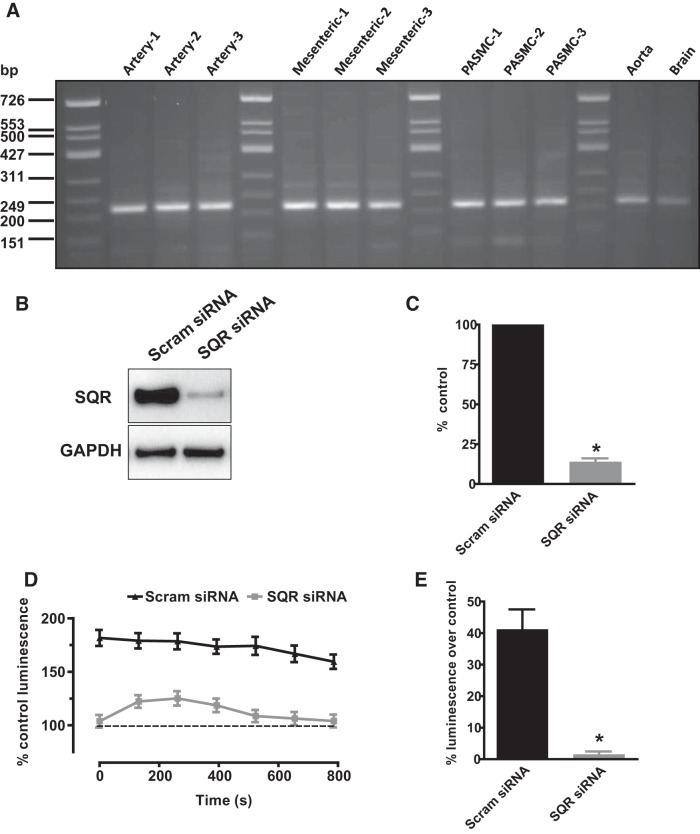
Evidence for a role of sulfide-quinone oxoreductase (SQR) in sulfide-induced reactive oxygen species (ROS) production. *A*: expression of SQR mRNA in homogenates from segments of rat pulmonary artery, mesenteric artery, cultured pulmonary artery smooth muscle cells (PASMCs), aorta, and whole brain. *B*: example of immunoblot showing SQR protein bands in homogenates from PASMCs treated with either scrambled or anti-SQR small interfering RNA. *C*: mean effect of anti-SQR siRNA observed in 7 experiments of the type shown in *B*. *D*: an example of an experiment in which L-012 luminescence was recorded in cells treated with scrambled (*top set* of points) or anti-SQR siRNA (*bottom set* of points) is shown on the *left*. Recordings were made immediately after cells were incubated in PSS containing 1,000 μM Na_2_S for 10 min and washed twice with PSS. The dashed lines indicate the level of luminescence in cells not treated with sulfide (i.e., the control level), recorded in triplicate. *E*: values at each time point, expressed as %control, were averaged, and the mean results from 5 experiments carried out using the same protocol. *P < 0.05, scrambled siRNA vs. SQR si RNA.

To examine the role of SQR in the response to sulfide, we measured the effect of application of 1,000 μM sulfide on ROS production in cultured PASMCs transfected with siRNA to knock down this enzyme. Figure 13*A* illustrates that anti-SQR siRNA, but not scrambled siRNA, markedly diminished SQR protein expression in PASMCs.

As described in methods, the effect of a 10-min treatment of PASMCs with 1,000 μM sulfide on ROS levels, assessed using the fluorescent probe L012, was assessed in PASMCs treated with scrambled or anti-SQR siRNA. As depicted in Fig. 13, *B* and *C*, sulfide pretreatment caused a sustained increase in the L012 signal in the cells treated with scrambled siRNA; this was absent in cells in which SQR had been knocked down. Figure 13, *D* and *E*, illustrates that ROS production by PASMCs was almost abolished in PASMCs treated with anti-SQR as compared with scrambled siRNA.

## DISCUSSION

It has previously been shown that application of 1,000 μM NaHS to preconstricted rat PAs induces a unique triphasic effect on tension consisting of two phases of contraction separated by a relaxation (25). This response bears some resemblance to HPV in these arteries. Based on this similarity and other evidence, Olson et al. (27) proposed that HPV is triggered by an increase in the intracellular concentration of sulfide, which would be predicted to occur under hypoxic conditions. We have recently reported that antagonists of the enzymes that synthesize sulfide do not inhibit HPV, which argues against this model (30). The present study was carried out as a complementary approach to examining this hypothesis by determining to what extent the mechanisms underlying the sulfide response, which remain obscure, resemble those of HPV.

We used relatively high concentrations of the sulfide donor Na_2_S (500 or 1,000 μM) in most experiments because these evoked the HPV-like double contraction, with the second more sustained component of the sulfide response in these concentrations being similar in size to Ph2 of HPV (33) and also because high cellular levels of sulfide have been proposed to exist during hypoxia, but we also examined the effects of 30 μM Na_2_S because these are likely to be more physiologically relevant under normoxic conditions. When using high sulfide concentrations, we focused mainly on investigating the Ph2 contraction since its long duration implies it is more likely to be relevant to the sustained Ph2 of HPV observed in rat PAs (7).

**Fig. 14.**
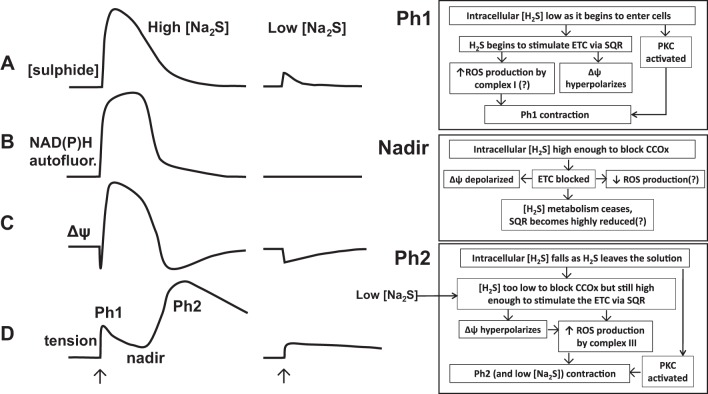
Proposed mechanisms of sulfide-induced contraction. Shown on the *left* and *middle* are the effects of applying (at the arrows) high (300–1,000 μM) and low (30 μM) Na_2_S concentrations to the myograph solution chamber on the solution sulfide concentration (*trace A*), NAD(P)H autofluorescence (*trace B*), mitochondrial membrane potential (*trace C*), and tension development (*trace D*) observed in rat pulmonary arteries. The 2 phases of contraction, separated by the intervening relaxation to a tension nadir, are indicated. One the right, the events observed or proposed to occur during each of these 3 phases of the response to high concentrations of sulfide are shown, as are their proposed relationships to each other. The events following the application of a low concentration of sulfide are proposed to be similar to those occurring during Ph2 of the response to high sulfide concentrations: metabolism of sulfide by SQR leading to electron transport chain (ETC) stimulation, hyperpolarization of ΔΨ, ROS production by complex 3, and contraction. No nadir of contraction occurs with 30 μM Na_2_S because the cellular sulfide concentration never gets high enough to block the ETC and therefore ROS production. In both high and low concentrations of sulfide, stimulation of PKC, likely caused by sulfide-induced thiol signaling, also enhances tension development throughout the response. CCOx, cytochrome-*c* oxidase.

As described below and illustrated in Fig. 14, we propose that both the Ph2 contraction at high Na_2_S concentrations and the monophasic contraction observed at lower concentrations are due to sulfide metabolism by SQR, resulting in a stimulation of the ETC that leads to an increased production of ROS by complex III.

### 

#### The onset of the Ph2 contraction occurs when the sulfide concentration falls to a level at which unblock of the ETC occurs and are associated with stimulation of the ETC.

It is important to appreciate that the [sulfide] in solutions open to the atmosphere decreases rapidly when sulfide is applied as a Na^+^ salt, as it has been in most in vitro studies (17). Our results show that this fall plays a critical role in shaping the biphasic nature of the response to ≥100 μM sulfide, such that the Ph2 contraction develops only when the [sulfide] in the solution has fallen to a low level.

The rate at which the [sulfide] in the solution fell varied between experiments, an effect that could to some extent be controlled by gassing the solution in the myograph chamber more or less vigorously. Using this approach, we found that the rate at which the solution [sulfide] fell was correlated with the delay before the Ph2 contraction developed (Fig. 8), such that the peak of the Ph2 contraction invariably occurred at a time when the [sulfide] had fallen into a narrow concentration range near 50 μM, regardless of how long it took for this to occur.

In separate experiments, simultaneous measurement of tension and NAD(P)H autofluorescence, the latter used as an indicator of ETC inhibition, demonstrated that the onset of the Ph2 contraction coincided with the fall in the autofluorescence signal evoked by ≥100 μM sulfide (Fig. 3), the abrupt nature of which was consistent with the requirement for three sulfide molecules to bind in order for cytochrome-a3 blockade to occur (38).

Taken together, the results shown in Figs. 3, 5 and 8 indicate that the Ph2 contraction occurred when the cytosolic [sulfide] fell sufficiently for complex IV to become unblocked. Although it could alternatively be argued that Ph2 was a delayed result of the previous ETC blockade, perhaps triggered by depolarization of ΔΨ and a consequent shift of Ca^2+^ from the mitochondria to the cytoplasm, the virtual simultaneity of the onset of Ph2 and the fall in NAD(P)H autofluoresence suggested that this was not the case. Moreover, the observation that applying low concentrations of Na_2_S (10 and 30 μM) caused a sustained monophasic contraction without any block of the ETC (as evidenced by the absence of any increase in NAD(P)H autofluorescence) implied strongly that sulfide-induced contraction was not dependent on block of the ETC or mitochondrial depolarization.

Instead the results indicated that sulfide-induced contraction was invariably associated with mitochondrial hyperpolarization. Higher concentrations of sulphide (300–1,000 μM) caused a triphasic change in ΔΨ, consisting of a very brief initial hyperpolarization, followed by a depolarization and then a second, prolonged hyperpolarization. This response was more prominent if the mitochondria were first depolarized by rotenone. Simultaneous recording of ΔΨ and tension in both the absence and presence of rotenone demonstrated that onset of the Ph2 contraction was closely associated with the second hyperpolarization of ΔΨ. Lower concentrations of sulfide (10 and 30 μM), which caused only a hyperpolarization of ΔΨ, evoked a monophasic contraction.

These results demonstrate that both the Ph2 contraction evoked after a delay following application of high [sulfide] and the immediate monophasic contraction caused by lower concentrations were associated with stimulation of the ETC. Although sulfide has long been known to block the ETC (9), with a reported IC_50_ in intact cells of several micromolar (e.g., Ref. 4), it has more recently become clear that at lower concentrations it stimulates the ETC. Elegant studies by Bouillard and colleagues (12, 19, 38) have established that this effect, which may predominate at low micromolar [sulfide], is due to its metabolism by a sulfide oxidation unit consisting of SQR, a dioxygenase and a sulfur transferase. SQR is reduced during this process and passes two electrons into the ETC, thereby increasing mitochondrial respiration. Stimulation of the ETC probably dominates in an even higher sulfide concentration range in intact arteries, as it has been shown (17) that [sulfide] up 100 μM increased mitochondrial O_2_ consumption in intact aorta.

Given that sulfide-induced contraction was associated with an index of ETC stimulation (hyperpolarization of ΔΨ), we reasoned that the Ph2 might depend, at least in part, on sulfide metabolism by SQR and the resulting increase in mitochondrial electron flow. It would be predicted that this process would be inhibited while the [sulfide] is high enough to block the ETC, but would then develop when the [sulfide] falls enough for unblock of complex IV to occur, thereby explaining why the Ph2 contraction is delayed until the offset of the NAD(P)H autofluorescence signal and the second hyperpolarization of ΔΨ, both of these events being indicative of a resumption of electron flow. This mechanism is also consistent with the immediate hyperpolarization of ΔΨ and the monophasic contraction observed upon the application of 10 and 30 μM sulfide. Similarly, the entry of electrons from SQR into the ETC at coenzyme Q (between complex I and III) would explain why the Ph2 contraction was attenuated by myxothiazol, which blocks electron entry into complex III, but not rotenone, which blocks complex I.

Generation of ROS by complex III has been identified as being responsible for initiating HPV (39, 41), and we propose that the interaction of H_2_S with SQR also increases ROS production by complex III. In particular, sulfide-induced hyperpolarization of ΔΨ would be predicted to increase ROS release by the mitochondria by increasing the lifetime of semireduced species such as ubisemiquinone, which can donate electrons to O_2_ to form superoxide, and also by increasing the driving force for superoxide to move into the mitochondrial intermembrane space where it can be converted to H_2_O_2_, which then enters the cytoplasm (13, 42). The block of the ETC during the period where the sulfide concentration is high could further promote the subsequent Ph2 contraction by preventing SQR oxidation and thereby rendering it highly reduced, thus setting the stage for it to fuel a complex III-mediated “burst” of ROS production when the [sulfide] falls enough to unblock cytochrome-a3.

This model is supported by the strong expression of SQR in rat PAs and PASMCs and the abolition by anti-SQR siRNA of the ROS production that occurred when PASMCs were treated with sulfide and then washed to allow the unblocking of complex IV. Moreover, the antioxidant TEMPOL strongly suppressed the Ph2 contraction (Fig. 9). As we have previously shown that ROS produce a Rho kinase-dependent constriction of these arteries (15), additional observations that were consistent with a role for ROS in the response to sulfide were its sensitivity to Y27632 and also the sulfide-induced activation of RhoA (Fig. 10). Additionally, Skovgaard and Olson (35) have demonstrated that the contraction of trout gills to sulfide was inhibited by mitochondrial blockers and the antioxidant diethyldithiocarbamate.

#### Role of PKC and NADPH oxidase.

Sulfide has been shown to cause a rapid activation of PKCε in cardiomyocytes (5), an effect that is thought to be involved in ischemic preconditioning (29). We observed that the nonselective PKC blocker Gö6983 attenuated both phases of the sulfide-induced contraction. Interestingly, the NOX inhibitor VAS2870 had a similar effect on the Ph2 contraction. PKC activation induces contraction in PAs and other arteries (40), and several PKC isoforms, including PKCε, stimulate NOX (20). We have recently shown that the sphingolipid sphingosylphosphorylcholine strongly potentiates PA contraction via a ROS-dependent pathway involving the activation of NOX1 by PKCε (34). Additionally, mitochondrial ROS produced from complex III have been shown to activate NOX by stimulating PKCε in PASMCs (32).

#### Role of Ca^2+^-elevating pathways in the sulfide response.

Although we focused mainly on the involvement of the mitochondria in the sulfide response, we also examined the potential involvement of several potential downstream Ca^2+^-elevating mechanisms. Nifedipine had no effect on either phase of the response to 1,000 μM sulfide, indicating the lack of involvement of voltage-gated Ca^2+^ channels. Conversely, ryanodine, at a concentration that blocks all three RyR isoforms, markedly suppressed the Ph1 and Ph2 contractions and also deepened the nadir of the intervening relaxation, suggesting that the major source of Ca^2+^ throughout the sulfide response was SR Ca^2+^ release via the RyR. Although dantrolene, which blocks RyR1 and 3 (14), also enhanced the relaxation, it had little effect on either phase of contraction, indicating that RyR2 probably plays the predominant role in sulfide-induced Ca^2+^ release. Interestingly, it has been demonstrated (23) that ROS induce Ca^2+^ release by the RyR2 receptor in PASMCs by causing its dissociation from FKBP12.6, and there is evidence that SR expressing RyR2 is dispersed widely within PASMCs, unlike RyR1 and 3, which demonstrate a subplasmalemmal and perinuclear distribution, respectively (6, 11).

As described above, both the Ph2 contraction and the monophasic response to 30 μM sulfide were associated with mitochondrial hyperpolarization. Furthermore, both responses were similarly inhibited by myxothiazol, Gö6983, and TEMPOL, implying that both responses share a common mechanisms: stimulation of the ETC, mitochondrial ROS production, and stimulation protein kinase C. However, ryanodine, which attenuated the Ph2 contraction to 1,000 μM sulfide, did not affect the response to 30 μM sulfide. A possible explanation for this is that whereas ROS production by 1,000 μM sulfide is sufficient to cause SR Ca^2+^ release, this is not the case with the lower concentration.

#### Mechanisms of the Ph1 contraction.

The Ph1 contraction to 1,000 μM Na_2_S occurred during a period in which the effects of sulfide on the mitochondria were in very rapid flux, making it difficult to determine the extent to which these events were playing a role in evoking this contraction. However, this contraction was slightly but significantly attenuated by rotenone. We therefore speculate that Ph1 could have been due in part to increased ROS production by complex I owing to a reverse flow of electrons entering the ETC as the very high concentration of sulfide was metabolized by SQR, although more work will be necessary to determine the validity of this idea. On the other hand, the lack of effect of myxothiazol on Ph1 indicates that ROS production by complex III was not involved. Although it is therefore unclear whether the mitochondria were involved in Ph1, its unambiguous block by Gö6983 and ryanodine indicates that this contraction was dependent on both PKC and RyR-mediated Ca^2+^ release from the SR. In any case, the fact that Ph1 contraction was occurring during a period when the cellular sulfide concentration in cells was almost certainly very high means that its physiological relevance is questionable. It is also possible that the transient increase in pH during the first several minutes after the application of 300–1,000 μM Na_2_S might have affected the amplitude of Ph1, most likely by attenuating it as alkalinization has been shown to relax PAs (10).

#### Block of l-cysteine-induced contraction by myxothiazol and Gö6983.

l-cysteine, the main cellular source of sulfide, causes an increase in tension in PGF_2α_-preconstricted PAs that is prevented by PAG, a blocker of the sulfide-synthesizing enzyme cystathionine-γ-lyase. Unlike Na_2_S, l-cysteine elicits a monophasic and sustained increase in tension, as well as a linear release of sulfide from liver, both effects implying that its application causes a steady increase in the cellular [sulfide] (30). As shown in Fig. 11*B*, both myxothiazol and Gö6983 strongly suppressed the increase in tension evoked by l-cysteine over a wide range of concentrations. This suggests that the mechanisms we have described for the contraction caused by Na_2_S may also occur with smaller and more sustained increases in the cellular [sulfide] that are likely to be more physiologically relevant.

#### Sulfide and HPV: similarities and differences.

A comparison of the mechanisms of the Ph2 contractile response to sulfide in rat PAs described in this study and those we have previously reported for HPV in the same arteries (7) reveals a number of similarities but two important differences. The sustained contractile responses to sulfide and hypoxia are both sensitive to blockade by ryanodine, Y-27632, Gö6983, TEMPOL, and myxothiazol and both insensitive to nifedipine. On the other hand, the Ph2 sulfide-induced contraction is inhibited by the NADPH oxidase blocker VAS 2870, which has no effect on HPV (7). Moreover, HPV is abolished by 1 μM rotenone (22, 41), which however had no significant effect on the sustained phase of the sulfide response.

The mechanisms by which hypoxia constricts PAs remain controversial, but there is substantial evidence that HPV is triggered by increased ROS production by complex III (22, 41), leading to SR Ca^2+^ release and the opening of store operated Ca^2+^ channels, as well as activation of Rho kinase. In this case, a substantial degree of similarity between the responses to sulfide and hypoxia would be predicted if sulfide also increases ROS production via complex III. However, the observation that rotenone and VAS 2870 exert differential effects on HPV and the sulfide response argues against the concept that sulfide may mediate HPV via this pathway, and, more generally, does not support a crucial role for sulfide in HPV.

#### Conclusion.

In summary, our observations offer a coherent explanation for the unique biphasic contraction induced in PAs by high concentrations of sulfide, while at the same time suggesting that a smaller but more sustained increase in cellular [sulfide] may activate similar pathways. Given the apparently widespread expression of SQR in the vasculature, and since ROS tend to exert complex effects on contraction in systemic as well as PAs (e.g., Ref. 36), the mechanisms we describe may have a wider implications for the vascular system as a whole.

## GRANTS

J. Prieto-Lloret, V. Snetkov, and Y. Shaifta were supported by Wellcome Trust Programme Grant 087776 (to J. P. Ward and P. I. Aaronson). M. J. Connolly and C. E. MacKay were supported by PhD Studentships from British Heart Foundation Grants FS/05/117/19967 (to P. I. Aaronson) and FS/12/43/29608 (to G. A. Knock). I. Docio was supported by an Erasmus Traineeship.

## DISCLOSURES

No conflicts of interest, financial or otherwise, are declared by the authors.

## AUTHOR CONTRIBUTIONS

J.P.-L., V.A.S., Y.S., I.D., M.J.C., C.E.M., G.A.K., and P.I.A. performed experiments; J.P.-L., V.A.S., Y.S., M.J.C., and P.I.A. analyzed data; J.P.-L., V.A.S., J.P.W., and P.I.A. interpreted results of experiments; J.P.-L., V.A.S., and Y.S. prepared figures; J.P.-L., V.A.S., J.P.W., and P.I.A. edited and revised manuscript; J.P.-L., V.A.S., Y.S., I.D., M.J.C., C.E.M., G.A.K., J.P.W., and P.I.A. approved final version of manuscript; P.I.A. conceived and designed research; P.I.A. drafted manuscript.
